# Exploring the prevalence and chest CT predictors of Long COVID in children: a comprehensive study from Shanghai and Linyi

**DOI:** 10.3389/fped.2024.1420196

**Published:** 2024-08-07

**Authors:** Yong Yin, Guijun Yang, Na Wang, Mei Zeng, Hejun Jiang, Shuhua Yuan, Jinhong Wu, Jing Zhang, Juan Cui, Guifang Zhou, Xin Yang, Yunqin Zhang, Zhichao Sun, Jiajun Yuan, Jilei Lin, Jiande Chen, Mingyu Tang, Jing Chen

**Affiliations:** ^1^ Department of Respiratory Medicine, Linyi Maternal and Child Health Care Hospital, Linyi Branch of Shanghai Children’s Medical Center, Shanghai Jiao Tong University School of Medicine, Linyi, Shandong, China; ^2^ Department of Respiratory Medicine, Shanghai Children’s Medical Center, Shanghai Jiao Tong University School of Medicine, Shanghai, China; ^3^ Shanghai Children’s Medical Center Pediatric Medical Complex (Pudong), Shanghai, China; ^4^ Pediatric AI Clinical Application and Research Center, Shanghai Children’s Medical Center, Shanghai, China; ^5^ Shanghai Engineering Research Center of Intelligence Pediatrics (SERCIP), Shanghai, China; ^6^ Child Health Advocacy Institute, China Hospital Development Institute, Shanghai Jiao Tong University, Shanghai, China; ^7^ Medical Department of Shanghai Children’s Medical Center, Shanghai Jiao Tong University School of Medicine, Shanghai, China

**Keywords:** long COVID, respiratory tract infections, children, CT abnormalities, predictive factors

## Abstract

**Introduction:**

COVID-19 constitutes a pandemic of significant detriment to human health. This study aimed to investigate the prevalence of Long COVID following SARS-CoV-2 infection, analyze the potential predictors of chest CT for the development of Long COVID in children.

**Methods:**

A cohort of children who visited the respiratory outpatient clinics at Shanghai Children's Medical Center or Linyi Maternal and Child Health Care Hospital from December 2022 to February 2023 and underwent chest CT scans within 1 week was followed up. Data on clinical characteristics, Long COVID symptoms, and chest CT manifestations were collected and analyzed. Multivariate logistic regression models and decision tree models were employed to identify factors associated with Long COVID.

**Results:**

A total of 416 children were included in the study. Among 277 children who completed the follow-up, the prevalence of Long COVID was 23.1%. Chronic cough, fatigue, brain fog, and post-exertional malaise were the most commonly reported symptoms. In the decision tree model for Long COVID, the presence of increased vascular markings, the absence of normal CT findings, and younger age were identified as predictors associated with a higher likelihood of developing Long COVID in children. However, no significant correlation was found between chest CT abnormality and the occurrence of Long COVID.

**Discussion:**

Long COVID in children presents a complex challenge with a significant prevalence rate of 23.1%. Chest CT scans of children post-SARS-CoV-2 infection, identified as abnormal with increased vascular markings, indicate a higher risk of developing Long COVID.

## 1 Introduction

Coronavirus Disease 2019 (COVID-19) is a spectrum of diseases caused by the Severe Acute Respiratory Syndrome Coronavirus 2 (SARS-CoV-2) (1), which has been prevalent for over four years. In December 2022, China lifted epidemic prevention policies related to SARS-CoV-2 infection, leading to a significant number of infections. Although it has been recognized that some complications, including fatigue, discomfort, alterations in smell and taste, difficulty breathing, and cognitive impairments, may occur in the short to medium term following a confirmed diagnosis of COVID-19 (2–4), research has primarily focused on the adult population. Studies on the long-term complications in children following SARS-CoV-2 infection are comparatively limited, and there are differences in symptoms between children and adults (5, 6). Ha et al. found that Long COVID in children is associated with a higher prevalence of mental health problems compared to adults (7). Just as with the disease itself, there is currently heterogeneity in the definition of Long-term effects of COVID-19 (Long COVID), with no unified definition established to date (8–16). Definitions of the long-term syndrome still involve the duration of symptoms, clusters, or syndromes, or a combination thereof, with various descriptions existing.

During the acute phase of COVID-19, chest Computed Tomography (CT) manifestations typically include ground glass opacification, predominantly in the lower lobes, which may progress to more extensive areas of consolidation (17). Some cases may evolve into fibrosis, characterized by a reticular pattern, traction bronchiectasis, and pulmonary parenchymal bands (18). There is not yet a standard unified description of chest CT findings in children during the acute phase of COVID-19, but some literature suggested that the manifestations in children and adults are similar, primarily featuring ground glass opacifications and consolidations (19–24). However, there is currently a lack of research verifying the relationship between acute-phase chest CT manifestations and subsequent long-term complications in children following SARS-CoV-2 infection.

In this study, we collected electronic health record information and chest CT results of children with SARS-CoV-2 infection who visited the pediatric respiratory outpatient clinics of Shanghai Children's Medical Center or Linyi Maternal and Child Health Care Hospital from December 2022 to February 2023. We conducted telephone follow-up interviews from August 2023 to September 2023 to gather information on Long COVID symptoms in these children. Our aim was to investigate the incidence risk of Long COVID in children, study the enduring impact of the virus on children's bodies, and explore whether certain chest CT manifestations during the acute phase of COVID-19 could predict the occurrence of Long COVID.

## 2 Methods

### 2.1 Study design

**Figure 1:**
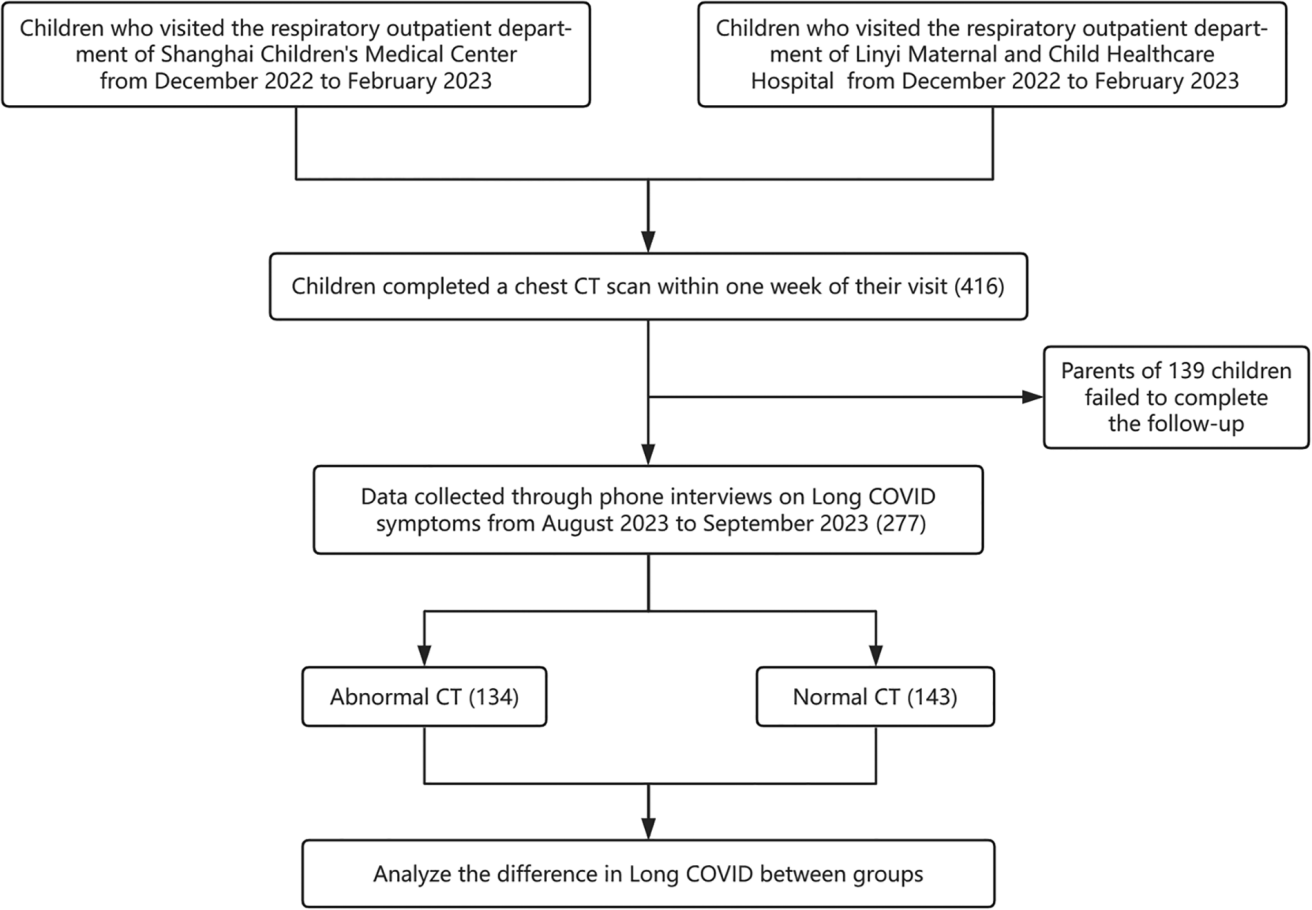
Flowchart illustrating the study design and participant selection process for the long COVID investigation in pediatric patients.

The study design and workflow are depicted in Figure 1. We selected children who visited the respiratory outpatient clinics at Shanghai Children's Medical Center or Linyi Maternal and Child Health Care Hospital from December 2022 to February 2023. The required sample size for this observational study was determined based on the expected prevalence of Long COVID, previously estimated at 9.5% (25). The calculation utilized the standard formula for estimating a proportion within a finite population with a designated precision:

$$n=\left(\frac{Z^{2}\times P(1-P)}{E^{2}}\right)$$

In this equation, *n* represents the necessary sample size, *Z* corresponds to the *Z*-score associated with a 95% confidence level (1.96), P denotes the anticipated prevalence rate, and E is the margin of error, which was predetermined at 5%. The calculation indicated a minimal requirement of approximately 132 participants to ensure sufficient statistical power to detect the stated prevalence with the intended precision. Anticipating potential issues such as participant dropout and incomplete data, an additional 20% was added to the initially calculated figure to mitigate the risk of underpowering the study. Thus, the target recruitment was adjusted to approximately 158 participants. Basic information such as gender, age, history of allergic rhinitis, asthma, and other relevant details were obtained from the outpatient electronic medical record system. Follow-up was conducted via telephone interviews from August 2023 to September 2023. The follow-up focused on monitoring the duration of symptoms within six months post-SARS-CoV-2 infection, including inquiries about the use of inhaled steroids, the number of fever episodes, respiratory infections, wheezing episodes, pneumonia cases, hospital admissions, and reinfection with SARS-CoV-2.

### 2.2 Inclusion and exclusion criteria

Inclusion Criteria: (1) Pediatric participants aged <18 years, male or female. (2) Visited the respiratory specialty outpatient clinics at Shanghai Children's Medical Center or Linyi Maternal and Child Health Care Hospital from December 2022 to February 2023. (3) Completed a chest CT scan within one week of the visit. (4) Diagnosed with SARS-CoV-2 infection or suspected cases. (5) Had complete outpatient electronic medical records. (6) Agreed to participate in the telephone follow-up. Exclusion Criteria: (1) Participants who deceased after contracting COVID-19. (2) Pediatric patients with congenital heart disease excluding postoperative cases, immunodeficiency, hematologic malignancies, neuromuscular disorders, genetic metabolic disorders, or other severe underlying conditions. (3) Inability of parents or guardians to provide accurate responses to follow-up questions during telephone follow-up. During the telephone follow-up, we reconfirmed with the parents whether the visit to the respiratory department from December 2022 to February 2023 was due to COVID-19.

### 2.3 Long COVID symptoms

Given the absence of a clear definition for Long COVID in children, we adopted the criteria for Long COVID in adults and children reported in the literature (16, 26–28) and integrated them with clinical experience in the diagnosis and treatment of pediatric COVID-19. These symptoms include olfactory or gustatory disorders, post-exertional malaise, chronic cough, brain fog (concentration or memory decline), dry mouth or thirst, palpitations, chest pain, fatigue, headache or dizziness, gastrointestinal symptoms, abnormal movements, anxiety, depression, sleep disorders, muscle pain, joint pain, sore throat, and rash. The World Health Organization (WHO) defines “Post COVID-19 Condition” as symptoms occurring in individuals with probable or confirmed COVID-19 infection three months after the onset of COVID-19, lasting for at least two months, and without an alternative diagnosis. For more information, please refer to the WHO website (https://www.who.int/zh/news-room/questions-and-answers/item/coronavirus-disease-(covid-19)-post-covid-19-condition). The Centers for Disease Control and Prevention (CDC) defines it as symptoms or health problems continuing four weeks after the onset of COVID-19. For more information, please refer to the CDC website (https://www.cdc.gov/coronavirus/2019-ncov/long-term-effects/). The United Kingdom uses a 12-week timeframe. For more information, please refer to the NHS website (https://www.nhs.uk/conditions/covid-19/long-term-effects-of-covid-19-long-covid/). In our study, we defined any symptoms persisting or emerging more than four weeks after acute SARS-CoV-2 infection in children as Long COVID symptoms.

### 2.4 Definition of chest CT abnormalities

Based on the literature (17–24) related to chest CT findings after SARS-CoV-2 infection in children, we defined chest CT abnormality 1 as ground glass opacities, patchy shadows, pulmonary nodules, centrilobular nodules, halo or reverse halo signs, consolidation (or air-space consolidation), bronchial inflation sign, crazy-paving pattern, white lung, reticular pattern or interstitial lung disease and interstitial changes.

Considering the less distinct typical chest CT manifestations in pediatric COVID-19 (29), prevalent chest CT abnormalities, such as ground glass opacities, patchy shadows, consolidation, pulmonary emphysema, cord-like shadows, pulmonary nodules, centrilobular nodules, bronchial inflation sign, tree-in-bud appearance, reticular pattern or interstitial lung disease, septal thickening, interstitial changes or abnormalities, halo or reverse halo signs, crazy-paving pattern, pleural effusion, reverse halo sign, pneumothorax, lung bullae, air trapping, bronchiectasis, increased vascular markings, bronchial wall thickening, necrotizing pneumonia, lung abscess, atelectasis, white lung and localized pleural thickening were defined as chest CT abnormality 2.

### 2.5 Data analysis and statistics

All statistical analyses were performed using the statistical package R version 4.3.2 (http://www.R-project.org, The R Foundation). Continuous variables were presented as means ± standard deviation (SD). Categorical variables were analyzed using the χ^2^ test or Fisher's exact test, as appropriate. A multivariate logistic regression model was utilized to assess the relationship between CT abnormality and Long COVID. Variables for the regression model were selected based on their clinical relevance and previous findings in the literature (30). Model I was adjusted for age, allergic rhinitis, asthma, and coinfections. Model II was adjusted for age, gender, eczema/atopic dermatitis, allergic rhinitis, asthma, food allergies, coinfections, underlying medical conditions, and the use of inhaled corticosteroids within 1 month. Additionally, based on Model I, subgroup analyses were conducted with the allergic rhinitis, asthma, and coinfection as the grouping variables. Furthermore, decision tree analysis was conducted to investigate the association between each individual chest CT manifestation and Long COVID. The complexity parameter (CP) for pruning the decision tree was chosen to avoid overfitting and to ensure the model's generalizability, with a value of CP = 0.01. This value was selected based on common practices and recommendations in the literature (31), which suggest that CP = 0.01 balances model complexity and predictive accuracy effectively. The sensitivity and specificity of the decision tree model were assessed using receiver operating characteristic (ROC) curve analysis. In the analysis, *P*-values less than 0.05 were considered statistically significant.

## 3 Results

### 3.1 Study participants and baseline characteristics

**Figure 2:**
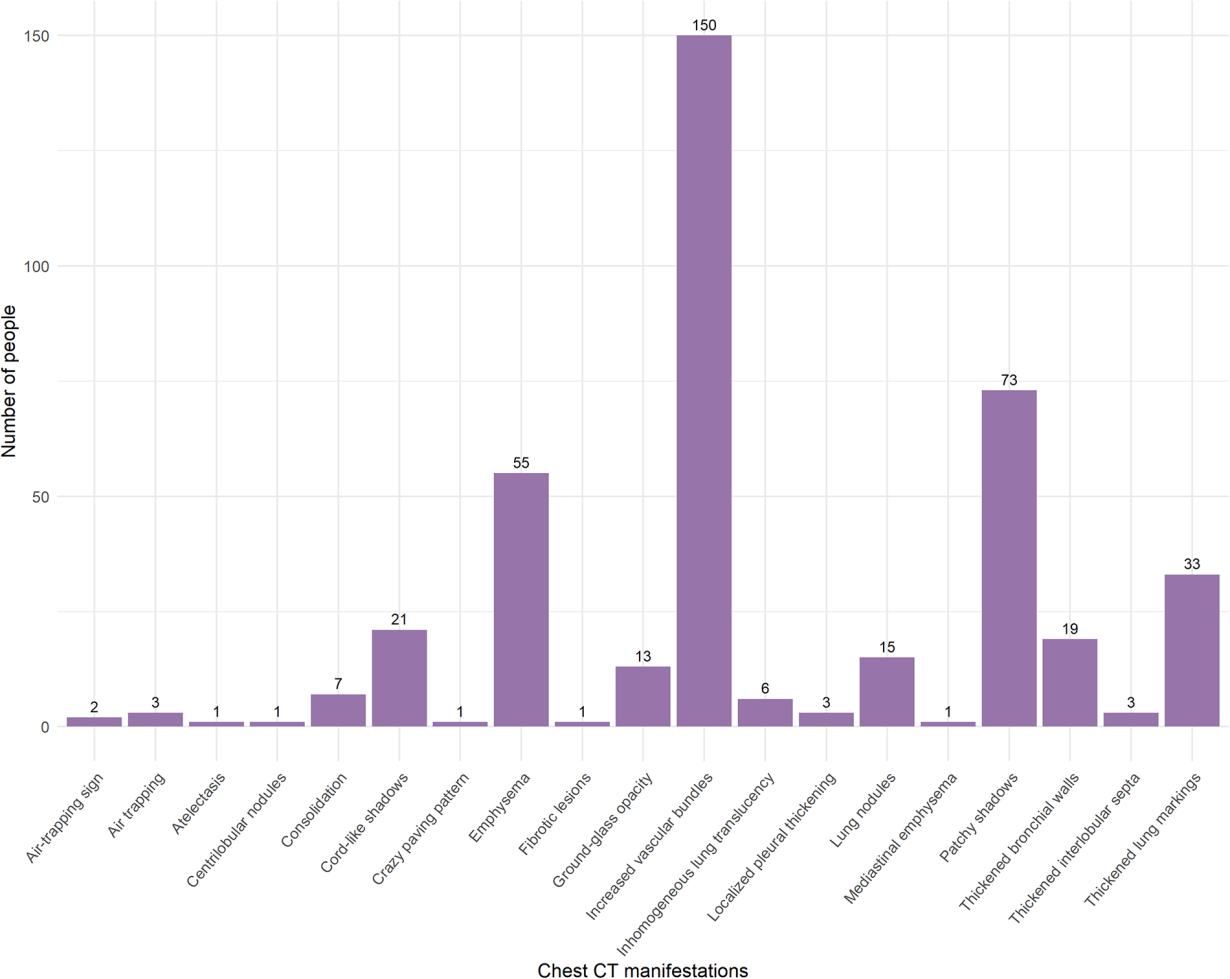
Frequency of chest CT manifestations in pediatric patients after SARS-CoV-2 infection.

**Figure 3:**
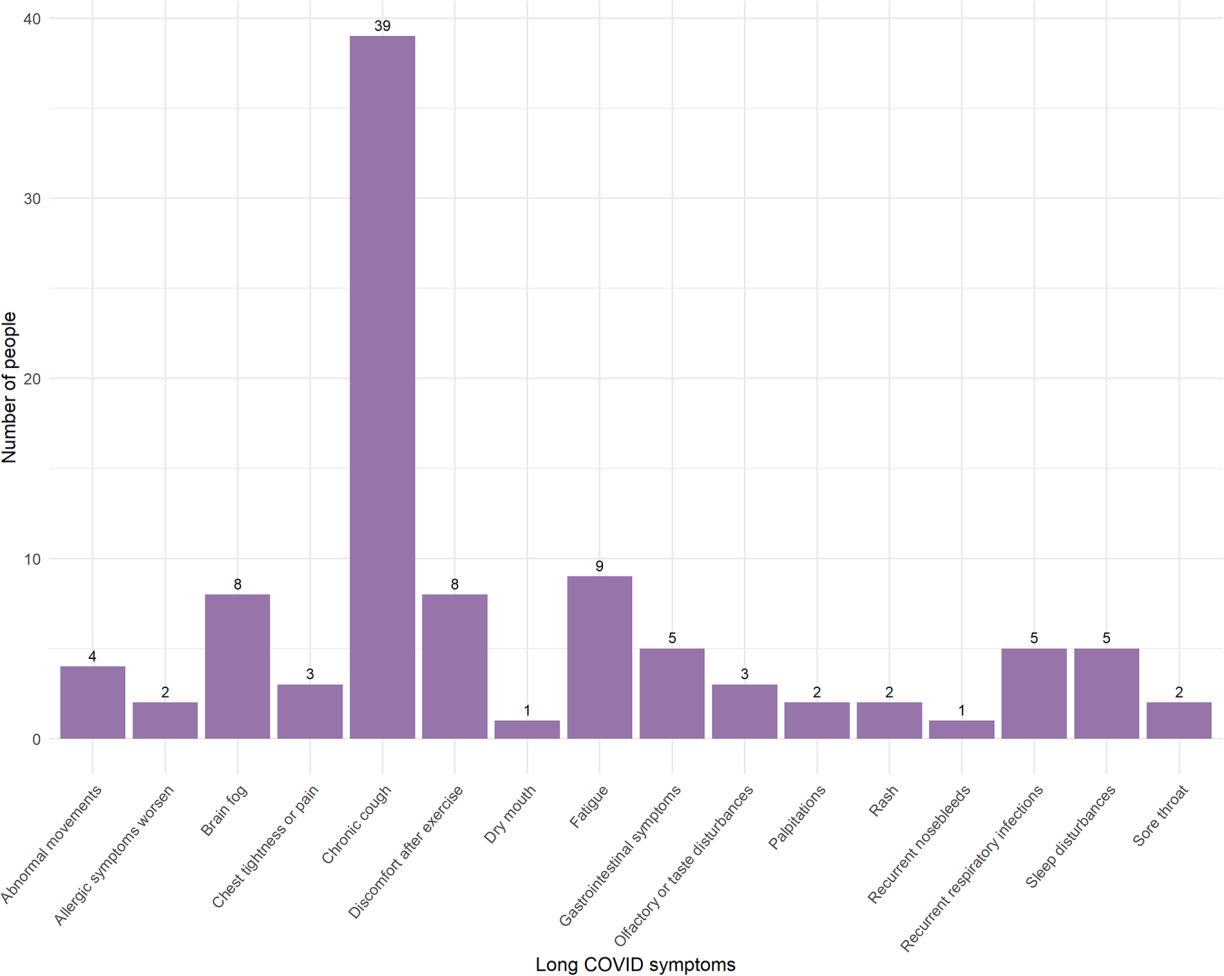
Frequency of long COVID symptoms reported by pediatric patients post-SARS-CoV-2 infection.

A total of 416 children were included in the study, 277 completed the follow-up. The characteristics of the study population are presented in Table 1. This study included participants under 18 years of age, with an average age of 6.14 ± 4.12 years; 62.09% were boys, and 37.91% were girls. There were a total of 25 patients with underlying medical conditions. Specifically, 3 participants had adenoid hypertrophy, 4 had food allergies, 3 had surgically treated or clinically insignificant atrial septal defects and ventricular septal defects, 2 had neurological disorders, 4 had chronic pulmonary diseases, 1 had a congenital pulmonary malformation that had been surgically corrected, 4 had endocrine disorders, 1 had a hematologic disorder and had undergone bone marrow transplantation, and 3 had renal diseases (These 25 participants did not meet the exclusion criteria.). The most common chest CT manifestation was increased vascular markings (Figure 2). Children with abnormal CT signs were significantly younger and were more likely to having coinfection. But they were less likely to suffer from allergic rhinitis. Figure 3 shows that chronic cough was the most frequently reported Long COVID symptom among children, reported by 39 individuals, while dry mouth and recurrent nosebleeds were the least frequent, with 1 report each. In this study, 143 out of 277 (51.6%) children exhibited abnormal chest CT findings after being infected with SARS-CoV-2. Long COVID symptoms were reported in 64 out of 277 cases (23.10%).

**Table 1:** Characteristics of the study population based on chest CT abnormality 2 (No or Yes).

	Statistics	No	Yes	Test value	*P*
Age (years), mean ± SD	6.14 ± 4.72	7.98 ± 3.90	4.41 ± 4.78	6.813	<0.001
Gender, *n* (%)					
Male	172 (62.09)	83 (61.94)	89 (62.24)	0	1
Female	105 (37.91)	51 (38.10)	54 (37.76)		
Eczema/Atopic dermatitis, *n* (%)					
No	208 (75.09)	100 (74.63)	108 (75.52)	0.001	0.9732
Yes	69 (24.91)	34 (25.37)	35 (24.48)		
Allergic rhinitis, *n* (%)					
No	181 (65.34)	69 (51.49)	112 (78.32)	20.819	<0.001
Yes	96 (34.66)	65 (48.51)	31 (21.68)		
Asthma, *n* (%)					
No	229 (82.67)	107 (79.85)	122 (85.31)	1.086	0.2975
Yes	48 (17.33)	27 (20.15)	21 (14.69)		
Food allergies, *n* (%)				0.187	0.6657
No	234 (84.48)	115 (85.82)	119 (83.22)		
Yes	43 (15.52)	19 (14.18)	24 (16.78)		
Underlying medical conditions, *n* (%)					
No	252 (90.97)	118 (88.06)	134 (93.71)	2.043	0.1529
Yes	25 (9.03)	16 (11.94)	9 (6.29)		
Coinfection, *n* (%)					
No	235 (84.84)	122 (91.04)	113 (79.02)	6.868	<0.05
Yes	42 (15.16)	12 (8.96)	30 (20.98)		
Using inhaled corticosteroids within 1 month					
No	252 (90.97)	119 (88.81)	133 (93.01)	1.019	0.3127
Yes	25 (9.03)	15 (11.19)	10 (6.99)		
Long COVID					
No	213 (76.90)	95 (70.90)	118 (82.52)	4.625	<0.05
Yes	64 (23.10)	39 (29.10)	25 (17.48)		

### 3.2 Factors associated with the occurrence of long COVID

**Figure 4:**
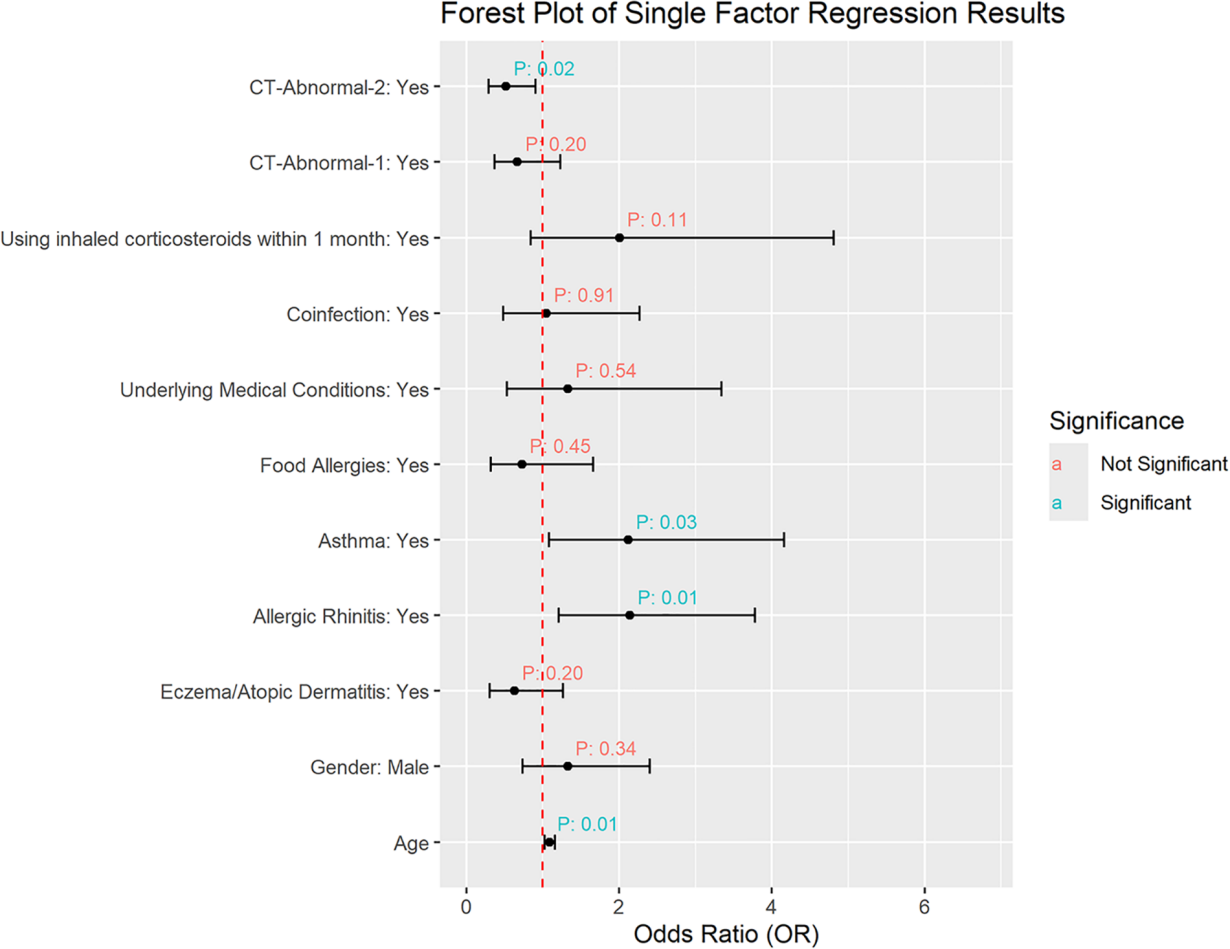
Factors associated with the occurrence of long COVID.

The results of the univariate regression can be seen in Figure 4. It shows that age, allergic rhinitis, and asthma are correlated with the occurrence of Long COVID. Children diagnosed with allergic rhinitis and asthma, as well as those of older age, showed increased susceptibility to developing Long COVID. Meanwhile, gender, eczema/atopic dermatitis, food allergies, underlying medical conditions, coinfection, and the use of inhaled corticosteroid within 1 month before the chest CT scan were not associated with the occurrence of Long COVID. Incorporating the baseline characteristics, age, allergic rhinitis, asthma, and coinfection were considered as the primary covariates in the multivariate regression analysis. Additionally, there was no association between chest CT abnormality definition 1 and Long COVID, while a negative correlation was observed between chest CT abnormality definition 2 and Long COVID, indicating that children with chest CT abnormalities according to the second definition had a lower probability of developing Long COVID.

### 3.3 Relationship between chest CT abnormality and long COVID

The results of the multivariate regression were presented in Table 2. After adjusting covariates for the models, neither Model I nor Model II showed a significant correlation between chest CT abnormality and the occurrence of Long COVID (*p* > 0.05). Subgroup analyses conducted with allergic rhinitis, asthma, and coinfection as grouping variables also showed no correlation between chest CT abnormality and Long COVID across all subgroups (Table 3).

**Table 2:** Relationship between chest CT abnormality and long COVID.

	L/N (%)	OR (95%CI) Model I	*P*	OR (95% CI) Model II	*P*
CT-abnormal-1					
No	45/176 (25.57)	1 (Ref)		1 (Ref)	
Yes	19/101 (18.81)	0.86 (0.45–1.67)	0.6692	0.92 (0.47–1.80)	0.801
CT-abnormal-2					
No	39/134 (29.10)	1 (Ref)		1 (Ref)	
Yes	25/143 (17.48)	0.66 (0.36–1.24)	0.197	0.68 (0.35–1.33)	0.230

Model I was adjusted for age, allergic rhinitis, asthma, and coinfections.

Model II was adjusted for age, gender, eczema/atopic dermatitis, allergic rhinitis, asthma, food allergies, coinfections, underlying medical conditions, and the use of inhaled corticosteroids within 1 month.

**Table 3:** Subgroup analysis of the relationship between chest CT abnormality and long COVID.

	CT-abnormal-1			CT-abnormal-2		
	OR (95CI)	*P*	P-interaction	OR (95CI)	*P*	P-interaction
Subgroup analysis stratified by allergic rhinitis						
No	1.15 (0.50–2.63)	0.7418	0.64	0.91 (0.40–2.10)	0.829	0.474
Yes	0.78 (0.24–2.58)	0.689		0.57 (0.21–1.53)	0.263	
Subgroup analysis stratified by asthma						
No	0.69 (0.31–1.51)	0.35	0.078	0.57 (0.27–1.21)	0.146	0.240
Yes	2.11 (0.56–8.02)	0.27		1.23 (0.36–4.23)	0.744	
Subgroup analysis stratified by coinfection						
No	0.86 (0.42–1.76)	0.680	0.8896	0.62 (0.31–1.21)	0.161	0.835
Yes	1.18 (0.21–6.76)	0.854		1.40 (0.23–8.35)	0.715	

Subgroup analyses were based on Model I.

### 3.4 Decision tree models of long COVID

**Figure 5:**
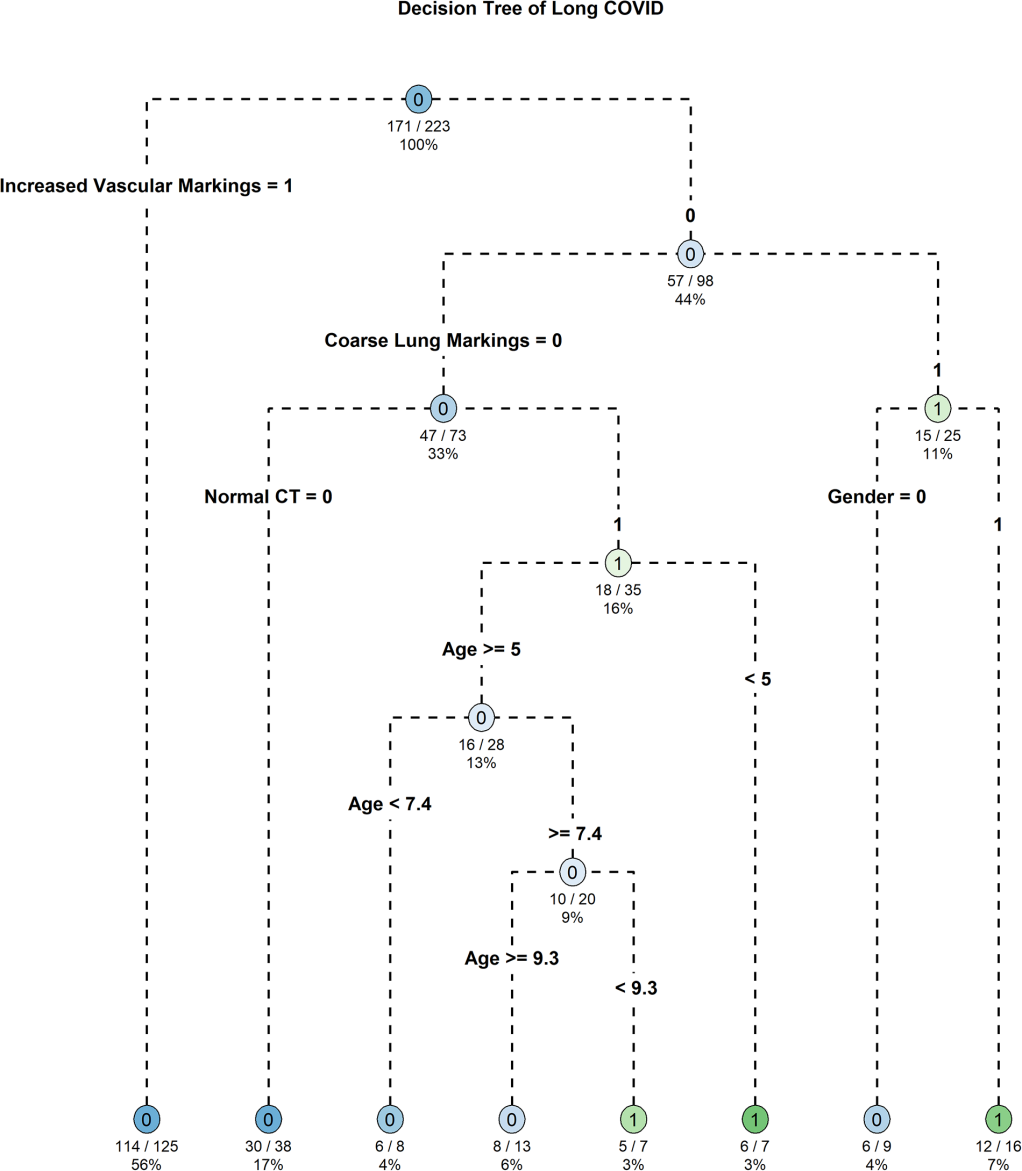
Decision tree model identifying Key predictors of long COVID in pediatric patients post-SARS-CoV-2 infection.

**Figure 6:**
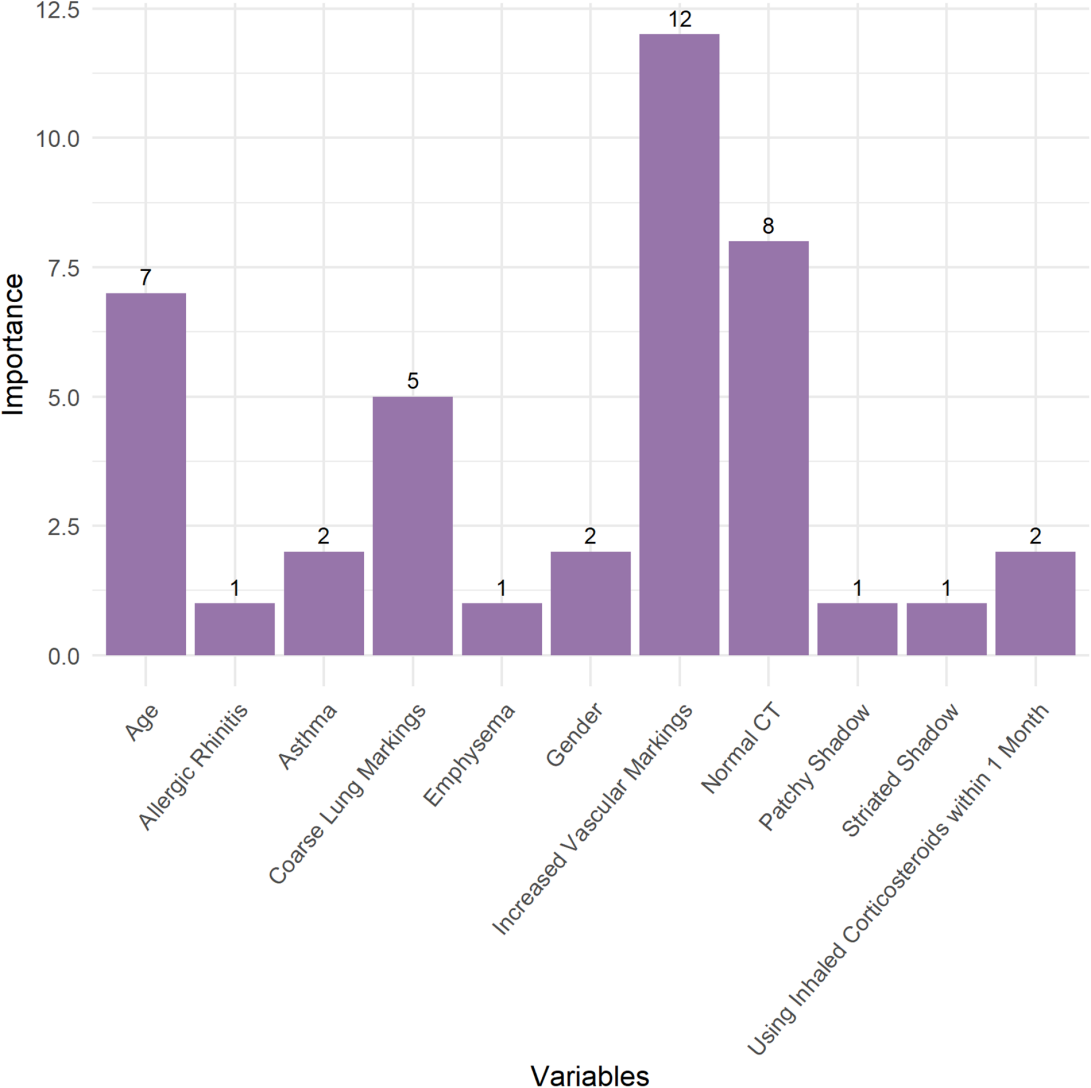
Variable importance of the decision tree for long COVID.

In this study, decision tree models were constructed to investigate the relationship between various chest CT manifestations, baseline characteristics, and the occurrence of Long COVID in children (Figure 5). The pruned decision tree model was developed based on a traindata comprising 223 observations (80%). The pruning of the tree was performed using a CP value of 0.01. The pruned decision tree model revealed several key variables associated with the likelihood of developing Long COVID. The most influential variables in the model included “Increased Vascular Markings”, “Normal CT”, “Age”, “Coarse Lung Markings”, “Asthma”, “Gender”, and “Using Inhaled Corticosteroids within 1 Month” (Figure 6). These variables were prioritized based on their importance in predicting the occurrence of Long COVID. Among the significant splits identified in the decision tree model, “Increased Vascular Markings” and “Normal CT” serve as the primary discriminators for predicting Long COVID. Specifically, when “Increased Vascular Markings” is true and “Normal CT” is false, it indicates a higher likelihood of children developing Long COVID. Furthermore, age was identified as a critical factor, with younger children demonstrating a higher propensity for developing Long COVID. The decision tree model was visualized in Figure 5, illustrating the hierarchical structure and significant predictors for Long COVID.

### 3.5 Model validation and ROC analysis

The pruned decision tree model demonstrated promising performance in the training dataset, achieving an accuracy of 79.37%. The confusion matrix revealed a sensitivity of 63.46% and a specificity of 84.21%. These findings suggest that the model effectively discriminates between children with and without Long COVID. In the testing dataset, the model achieved an accuracy of 74.07%. The confusion matrix showed a sensitivity of 58.33% and a specificity of 78.57%, indicating reasonable predictive performance on unseen data.

The ROC curves depict the discrimination performance of the pruned decision tree model in predicting Long COVID, evaluated using both the traindata (black) and testdata (red). 95% CI of traindata AUC: 0.722–0.869. 95% CI of testdata AUC: 0.648–0.921.

**Figure 7:**
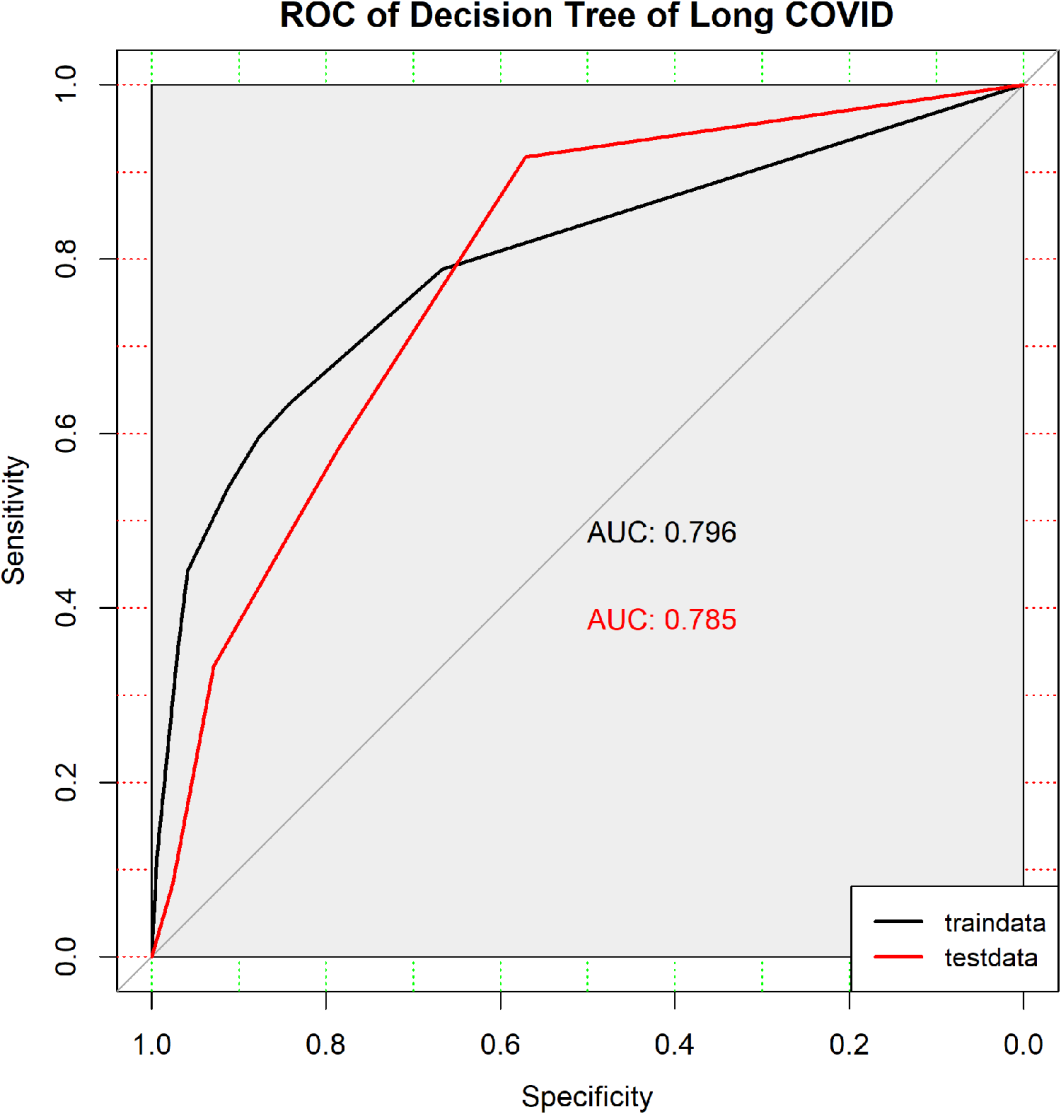
ROC curves for the decision tree model predicting long COVID in pediatric patients.

ROC curve analysis was conducted to further evaluate the discrimination ability of the pruned decision tree model. In the training dataset, the area under the curve was 0.796 (95% CI: 0.722–0.869), suggesting good discriminative power. Similarly, the area under the curve for the testing dataset was 0.7847 (95% CI: 0.648–0.921), indicating consistent performance in discriminating Long COVID. The ROC curves for both the training dataset and testing dataset are illustrated in Figure 7, providing visual representation of the model's performance in predicting Long COVID in children.

## 4 Discussion

In our study, two standards for defining chest CT abnormality were set. Standard one was based on previous literature, defining chest CT abnormality as those appearing after SARS-CoV-2 infection in children. Standard two defined any deviation from normal and some non-specific inflammatory responses as chest CT abnormality. We included all chest CT findings, including normal findings, and baseline characteristics in the decision tree model to predict the occurrence of Long COVID. The outcomes elucidated several key variables, including Increased Vascular Markings, Normal CT, Age, Coarse Lung Markings, Asthma, Gender, and Use of Inhaled Corticosteroids within one Month, which emerged as significant predictors of Long COVID. In pruned decision tree model of Long COVID, increased vascular markings, normal CT findings, and younger age suggested a higher likelihood of developing Long COVID in children.

However, our multivariate regression results showed no correlation between chest CT abnormality and the occurrence of Long COVID for either standard. Studies in adults have shown that chest CT severity scores and certain characteristic CT manifestations are associated with more severe acute-phase manifestations and long-term adverse outcomes (32–34). The absence of similar findings in children could be due to differences in physiology, immune response, and viral mechanisms between children and adults. Children's abnormal chest CT findings after COVID-19 infection are usually milder and recover more quickly (35), likely due to their more active and adaptable immune systems. Additionally, the types and functions of antibodies produced in children after SARS-CoV-2 infection differ from those in adults, which may help reduce viral persistence and lower the risk of Long COVID (36). Chronic cough, fatigue, brain fog (lack of concentration or memory decline), and post-exertional malaise were the most common Long COVID symptoms in children. This differs slightly from adults, where fatigue, sleep disorders, and difficulty breathing were primary symptoms. Follow-ups in adult hospitalized cohorts also showed persistent changes in lung structure and function (37). Our selection of participants from respiratory outpatient clinics may have contributed to a higher occurrence of respiratory-related symptoms, such as chronic cough. Nonetheless, our study indicated that Long COVID syndrome in children, like in adults, is a complex, multifaceted disease involving multiple systems (37–39).

In this study, the risk of Long COVID in children was 23.1%, which is higher compared to a study from the UK showing that the occurrence rate of Long COVID was 9.5% among people who received two doses of the COVID-19 vaccine and 14.6% among those who were unvaccinated (25). However, another meta-analysis showed that 45% of COVID-19 survivors, regardless of hospitalization status, experienced at least one unresolved symptom within an average follow-up time of 126 days. Moreover, the prevalence of persistent symptoms seems higher in post-hospitalization cohorts compared to non-hospitalized groups. This study excluded populations with specialist consultations and susceptible or high-risk groups (40). Current researches indicated that the risk of Long COVID varies significantly depending on ethnicity, age, vaccination status, and regional containment policies.

This study has several advantages. Firstly, it particularly focused on the children, who were often overlooked in COVID-19 related researches. This research contributed valuable information for understanding and managing Long COVID among children, aiding in the development of targeted interventions and public health strategies. Secondly, the study not only investigated the prevalence of Long COVID but also analyzed the association between chest CT manifestations and Long COVID, providing potential markers for early risk identification clinically. Thirdly, the research employed multivariate logistic regression models and decision tree models to identify factors associated with Long COVID, enhancing the rigor and reliability of the findings.

However, several limitations to this study should be noted. (1) Sample Size and Regional Representation: The research was primarily conducted at the Shanghai Children's Medical Center and Linyi Maternal and Child Health Care Hospital, involving 416 children with only 277 completing the follow-up. The relatively small sample size and the specific regional setting may not fully represent other regions or countries. (2) Selection and Recall Bias: The exclusion of children with severe underlying conditions, such as congenital heart disease, immunodeficiency, or neuromuscular disorders, may introduce selection bias. Additionally, the collection of symptoms of Long COVID was based on self-reporting by their parents, which could be influenced by recall bias, limiting the accuracy of the severity and duration of symptoms reported. (3) Disease Severity: The study did not consider the severity of the disease at the time of diagnosis, such as hospitalization status, which could influence the development and trajectory of Long COVID symptoms. Additionally, the study primarily included children who visited respiratory specialists, indicating a potentially more severe disease presentation with predominant respiratory symptoms. This focus may not accurately represent the broader pediatric population affected by COVID-19. (4) Follow-Up Period: The limited follow-up period of 6 months may not adequately capture the long-term outcomes and full spectrum of Long COVID in children. Addressing these limitations in future research would enhance the applicability and reliability of the study's conclusions.

Lastly, it is crucial to emphasize that while current evidence suggested a relatively lower risk of Long COVID in children, understanding the long-term effects of COVID-19 is still evolving. Comprehensive monitoring and research on the long-term health status of children diagnosed with COVID-19 are essential for fully understanding how the virus affects different age groups and for developing corresponding public health strategies (22).

## 5 Conclusion

Long COVID in children presents a complex challenge with a significant prevalence rate of 23.1%. While certain chest CT characteristics are predictive of Long COVID. These findings highlight the necessity for further research into Long COVID's impact on children and underscore the importance of developing targeted health management strategies for this population.

## Data Availability

The original contributions presented in the study are included in the article/Supplementary Material, further inquiries can be directed to the corresponding authors.
